# Dysphagia in Older Adults as a Geriatric Syndrome: From Disease‐Based Diagnosis to Mechanism‐Based Assessment

**DOI:** 10.1111/ggi.70841

**Published:** 2026-09-17

**Authors:** Hideaki Takahata

**Affiliations:** ^1^ Department of Rehabilitation Medicine Nagasaki University Hospital Nagasaki Japan

**Keywords:** dysphagia, geriatric syndrome, mechanism‐based assessment, older adults, rehabilitation

## Abstract

Dysphagia in older adults is commonly described using disease‐specific labels, such as post‐stroke dysphagia, Parkinson‐related dysphagia, or sarcopenic dysphagia. These labels are clinically useful, but they do not always explain the mechanisms that determine swallowing presentation or guide management in individual patients. Even within the same diagnostic category, one patient may present primarily with impaired neural control and delayed swallow initiation, whereas another may present with reduced muscular output, inefficient clearance, and fatigue. In daily practice, these mechanisms often overlap, and clinicians frequently adjust management according to the dominant and modifiable contributors rather than relying on the disease label alone. This narrative review reconsiders dysphagia in older adults as a multifactorial clinical condition that shares key characteristics of geriatric syndromes. It organizes the mechanisms contributing to dysphagia into clinically relevant domains, including neural control, muscle function, activity level, systemic reserve, and external modifiers. This perspective does not replace disease‐based diagnosis. Instead, it provides a structured way to make clinical reasoning more explicit, integrating physiological impairment, activity‐level limitation, and contextual factors. Understanding dysphagia in older adults as a geriatric syndrome may support more individualized, comprehensive, and multidisciplinary assessment and management in geriatric and rehabilitation practice.

## Graphical abstract

Dysphagia in older adults is often shaped by interacting neural, muscular, activity‐related, systemic, and care‐related factors rather than disease labels alone. This mechanism‐based perspective supports more explicit, individualized, and multidisciplinary assessment and management.

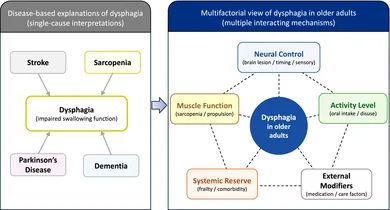

## 1 Introduction

Dysphagia is a common and clinically important problem in older adults. It is associated with malnutrition, dehydration, aspiration pneumonia, functional decline, prolonged hospitalization, institutionalization, and increased mortality [1, 2, 3]. Because eating and swallowing are closely linked to nutrition, dignity, social participation, and quality of life, dysphagia in later life is not merely a physiological impairment but a condition with broad clinical and social consequences [1, 4].

Traditionally, dysphagia has been classified according to disease‐specific diagnoses or presumed etiologies. Common examples include post‐stroke dysphagia, Parkinson‐related dysphagia, dysphagia associated with head and neck cancer, and sarcopenic dysphagia. These labels are clinically useful. They help clinicians recognize major causes, anticipate risks, communicate across disciplines, and initiate appropriate management.

However, in older adults, dysphagia is often more complex than a single diagnostic label suggests. Swallowing function depends on the interaction of neural control, muscle function, sensory input, airway protection, nutrition, activity, and the surrounding care environment [1, 2, 5]. Even when a disease label appears to explain the problem, the severity, persistence, and clinical consequences of dysphagia may be shaped by additional factors.

This complexity is familiar in geriatric practice. Older adults often live with reduced reserve and multiple vulnerabilities. Age‐related swallowing changes may remain compensated under stable conditions, but acute illness, hospitalization, fasting, reduced activity, or changes in medication and care can disturb this balance [1, 2, 6]. In such situations, dysphagia may emerge, persist, or worsen through the accumulation and interaction of several contributors.

These observations suggest that dysphagia in older adults can be considered within the broader framework of geriatric syndromes [1, 2]. Viewing dysphagia in this way does not require abandoning disease‐based diagnosis. It encourages clinicians to complement diagnostic labels with an assessment of the mechanisms and modifiable contributors that shape swallowing function.

The aim of this narrative review is not to propose a new disease entity or fixed classification system, but to reconsider dysphagia in older adults as a multifactorial geriatric syndrome and to clarify the clinical value of complementing disease‐based diagnosis with mechanism‐based assessment.

## 2 Multifactorial Nature of Dysphagia

### 2.1 Post‐Stroke Dysphagia: Reconsidering a Lesion‐Based Explanation

Post‐stroke dysphagia is one of the most established and clinically familiar forms of oropharyngeal dysphagia. Because dysphagia occurs after an identifiable cerebrovascular event, it is often understood primarily as a consequence of stroke‐related brain injury. This interpretation is clinically useful, particularly for recognizing neurogenic dysphagia and guiding early screening and management [7, 8]. Lesion location, lesion size, corticobulbar pathway involvement, brainstem damage, and bilateral hemispheric lesions are important factors in understanding swallowing impairment after stroke [9, 10, 11].

However, post‐stroke dysphagia also illustrates the limitations of a purely lesion‐based explanation. In clinical practice, the severity and course of swallowing dysfunction are not determined by lesion characteristics alone. Neurological severity, consciousness level, and functional impairment have been associated with dysphagia‐related outcomes or oral feeding status after stroke [12, 13, 14]. These factors, commonly assessed using measures such as the National Institutes of Health Stroke Scale and the Glasgow Coma Scale, indicate the overall burden of neurological impairment and the patient's ability to maintain alertness, voluntary control, posture, oral intake, and participation in swallowing rehabilitation.

In addition, severe stroke often requires acute management that reduces opportunities for swallowing activity, including temporary fasting, delayed oral intake, tube feeding, bed rest, and, in some cases, endotracheal intubation with mechanical ventilation [7, 8, 15]. Although these measures may be clinically necessary, prolonged reduction in swallowing activity and sensorimotor stimulation may contribute to secondary decline or delayed recovery. Thus, even post‐stroke dysphagia, which appears to have a relatively clear neurological cause, may not be fully explained by lesion‐based mechanisms alone. This example illustrates that the multifactorial nature of dysphagia can be observed even in conditions traditionally understood through a disease‐based framework.

### 2.2 Sarcopenic Dysphagia: Reconsidering a Purely Myogenic Interpretation

Sarcopenic dysphagia has been proposed as an explanatory concept for dysphagia in older adults without an obvious primary neurological or structural disease [16, 17, 18]. The concept has made an important contribution to geriatric dysphagia care by drawing attention to the role of generalized and swallowing‐related muscle loss, malnutrition, frailty, and reduced physical function. It has also provided a practical clinical framework that supports nutritional intervention, resistance‐based rehabilitation, and multidisciplinary management in older patients with impaired oral intake [17].

At the same time, recent reconsiderations of sarcopenic dysphagia suggest that caution is needed when interpreting this condition as a purely myogenic disorder. Diagnostic approaches vary across studies and clinical settings, particularly regarding the requirement for whole‐body sarcopenia, the exclusion of other causes, and the assessment of swallowing‐related muscle function [16, 19, 20]. As a result, patients with different clinical and pathophysiological backgrounds may be included under the same diagnostic label.

The coexistence of sarcopenia and dysphagia also does not necessarily establish a direct causal pathway from sarcopenia to swallowing dysfunction [16, 18, 21]. Dysphagia may contribute to reduced oral intake, malnutrition, and subsequent muscle loss, while sarcopenia may contribute to impaired swallowing. These bidirectional and temporal relationships complicate causal interpretation.

The physiological specificity of sarcopenic dysphagia remains difficult to establish. In many studies, dysphagia associated with sarcopenia has been evaluated using surrogate markers of swallowing‐related muscle function or oral intake measures. Measures such as tongue pressure and the Functional Oral Intake Scale (FOIS) are clinically useful [22, 23], but they do not necessarily demonstrate physiological impairment of oropharyngeal swallowing. Tongue pressure reflects one aspect of oral motor function and should not be equated with pharyngeal contraction or pharyngeal pressure, whereas FOIS primarily reflects functional oral intake status, including nil per os status, tube feeding dependence, and dietary modification. Recent work using fiberoptic endoscopic evaluation of swallowing (FEES) has also raised questions about the direct association between sarcopenia and physiological swallowing impairment [24]. These considerations suggest that dysphagia labeled as sarcopenic dysphagia may include heterogeneous clinical and pathophysiological backgrounds.

This perspective does not diminish the clinical significance of sarcopenic dysphagia. It suggests that sarcopenia should be understood as an important contributor within a broader multifactorial framework of dysphagia in older adults. Such a view allows muscle dysfunction to be recognized while also considering neural control, sensory function, activity level, systemic reserve, and external modifiers that may influence swallowing function in geriatric practice.

### 2.3 Dysphagia After Aspiration Pneumonia in Older Adults: A Prototypical Multifactorial Condition

Dysphagia after aspiration pneumonia in older adults provides a clinically familiar example in which single‐cause explanations are particularly difficult to apply. Unlike post‐stroke dysphagia, which may appear to have an identifiable neurological cause, or sarcopenic dysphagia, which is often interpreted through a muscle‐centered framework, swallowing dysfunction after aspiration pneumonia frequently emerges within a complex interaction of pre‐existing vulnerability and acute illness [25, 26].

Aspiration pneumonia in older adults is itself a multifactorial condition. It may occur in the context of impaired swallowing, poor oral health, reduced cough effectiveness, frailty, dependence in feeding, cognitive impairment, reduced mobility, and systemic illness [25, 26]. After the onset of pneumonia, additional factors may further compromise swallowing function. Systemic inflammation, fever, fatigue, respiratory compromise, fasting, bed rest, reduced oral intake, and decreased opportunities for swallowing use can all contribute to functional decline [25, 27, 28]. These factors may be particularly important in older adults with pre‐existing presbyphagia, frailty, sarcopenia, malnutrition, or reduced physiological reserve [1, 6, 16].

In this setting, dysphagia is not simply a pre‐existing cause of pneumonia, nor is pneumonia merely a consequence of dysphagia. The relationship may be bidirectional and dynamic [25, 26]. Pre‐existing swallowing vulnerability may increase the risk of aspiration pneumonia, while the pneumonia episode and its management may worsen swallowing function through disuse, systemic decline, respiratory dysfunction, and reduced alertness or activity [25, 27, 28]. External modifiers, including medication effects, fasting policies, feeding assistance, and care environment, may further influence this trajectory [26, 29].

Thus, dysphagia after aspiration pneumonia in older adults represents a prototypical multifactorial condition. It illustrates how swallowing dysfunction in geriatric practice may arise not from a single dominant cause but from the accumulation and interaction of vulnerabilities, acute stressors, systemic decline, and care‐related factors over time. This clinical scenario provides a bridge to understanding dysphagia in older adults as a geriatric syndrome.

## 3 Two Prototypical Mechanisms of Oropharyngeal Dysphagia

This section outlines two prototypical mechanisms of oropharyngeal dysphagia: neural control‐dominant dysphagia and muscle function‐dominant dysphagia. These prototypes are not intended to classify all patients into two exclusive categories. They serve as conceptual anchors for interpreting the mixed pathophysiological findings commonly observed in clinical practice. The contrast between these mechanisms is summarized in Table 1.

**TABLE 1:** Two prototypical mechanisms of oropharyngeal dysphagia.

Aspect	Neural control‐dominant dysphagia	Muscle function‐dominant dysphagia
Prototypical condition	Dysphagia in bulbar palsy	Dysphagia in Duchenne muscular dystrophy
Representative disorder	Lateral medullary infarction	Duchenne muscular dystrophy
Dominant mechanism	Impaired neural regulation	Reduced muscular output
Core problem	Swallow initiation, coordination, airway protection, and UES opening	Bolus preparation, propulsion, clearance, and endurance
Typical clinical features	Delayed or absent swallow triggering; impaired coordination; aspiration	Prolonged mealtime; chewing difficulty; fatigue
Instrumental findings	VFSS/FEES: delayed swallow initiation, premature bolus entry, impaired airway protection, aspiration, residue; VFSS/physiological studies: impaired UES opening or response	VFSS: oral phase problems, impaired bolus transport, post‐swallow residue, inefficient clearance; sEMG: reduced amplitude or prolonged activity with relatively preserved timing; tongue pressure: reduced
Conceptual role	Prototype for neural control‐dominant dysphagia	Prototype for muscle function‐dominant dysphagia
Limitation	Uncommon as an isolated pattern	Rare disease; uncommon as an isolated pattern

*Note:* Summarizes two prototypical mechanisms of oropharyngeal dysphagia: Neural control‐dominant dysphagia and muscle function‐dominant dysphagia. Dysphagia in bulbar palsy, represented by lateral medullary infarction, illustrates impaired neural regulation of swallow initiation, coordination, airway protection, and upper esophageal sphincter (UES) opening. Dysphagia in Duchenne muscular dystrophy illustrates reduced muscular output, impaired bolus preparation and propulsion, inefficient clearance, and fatigue. These prototypes are intended as conceptual anchors for interpreting clinical dysphagia and do not represent mutually exclusive categories.

Abbreviations: FEES, fiberoptic endoscopic evaluation of swallowing; sEMG, surface electromyography; UES, upper esophageal sphincter.; VFSS, videofluoroscopic swallowing study.

Bulbar palsy and Duchenne muscular dystrophy were selected not because they are the most common causes of dysphagia in older adults, but because they provide relatively distinct examples of neural control‐dominant and muscle function‐dominant mechanisms. In geriatric practice, pseudobulbar palsy and Parkinson‐related dysphagia are more commonly encountered examples of neurogenic dysphagia. These conditions may involve impaired voluntary control, corticobulbar pathway involvement, bradykinesia, rigidity, delayed swallow initiation, impaired airway protection, pharyngeal residue, cognitive impairment, and functional decline, and therefore often illustrate mixed mechanisms rather than a single isolated pathway [30, 31].

### 3.1 Neural Control‐Dominant Dysphagia: Bulbar Palsy

Bulbar palsy provides a representative example of neural control‐dominant oropharyngeal dysphagia. Lesions involving the lower cranial nerves, their nuclei, or related medullary swallowing networks disturb the sensorimotor control required for safe and efficient swallowing [32, 33]. In this context, dysphagia is characterized by impairment of swallow initiation, pharyngeal coordination, airway protection, and upper esophageal sphincter (UES) opening [32, 33, 34].

Videofluoroscopic studies have described delayed or absent swallow triggering, premature bolus entry into the pharynx, impaired coordination between bolus flow and airway protection, aspiration, and pharyngeal residue in bulbar dysphagia [33, 34]. Impaired bolus passage through the UES and absent or markedly impaired UES responses have also been demonstrated by videofluoroscopic, manometric, and electrophysiological studies [33, 34]. These findings indicate that a defining feature of bulbar dysphagia is disruption of neural control over swallow initiation, pharyngeal coordination, airway protection, and UES opening.

### 3.2 Muscle Function‐Dominant Prototype: Duchenne Muscular Dystrophy

Duchenne muscular dystrophy provides a representative example of muscle function‐dominant dysphagia. As a progressive neuromuscular disorder, it affects skeletal muscles involved in chewing, oral bolus control, pharyngeal contraction, and respiratory support [35, 36].

Clinically, dysphagia in this condition is associated with prolonged mealtime, difficulty chewing, reduced tongue and oral motor function, impaired bolus formation, pharyngeal residue, and reduced swallowing efficiency [35]. Videofluoroscopic studies have described oral phase problems, impaired bolus transport, post‐swallow residue, and inefficient pharyngeal clearance [35, 37]. These findings indicate that the bolus may not be adequately prepared, propelled, or cleared because of reduced performance of the oral and pharyngeal musculature.

Surface electromyography provides additional information on swallowing‐related muscle activity. Electromyographic findings suggest lower activity amplitude or prolonged swallowing‐related muscle activity, while the timing of muscle activation is relatively preserved [36]. Tongue pressure studies have also shown reduced tongue pressure, supporting reduced oral motor output as one component of dysphagia in this condition [38].

Taken together, these findings suggest that swallowing impairment in Duchenne muscular dystrophy is expressed mainly as reduced force generation, impaired bolus propulsion, fatigue, and inefficient clearance, with relatively preserved timing of muscle activation. Duchenne muscular dystrophy is therefore useful as a prototype for the muscle function‐dominant axis of oropharyngeal dysphagia.

### 3.3 From Prototypes to Mixed Mechanisms in Clinical Dysphagia

Bulbar palsy and Duchenne muscular dystrophy are useful prototypes because they illustrate two contrasting mechanisms of oropharyngeal dysphagia. Lateral medullary infarction is a representative cause of bulbar palsy, in which impaired neural control affects swallow initiation, airway protection, and upper esophageal sphincter opening [33, 34]. Duchenne muscular dystrophy represents a muscle function‐dominant pattern, characterized by reduced muscular output, impaired bolus propulsion, fatigue, and inefficient clearance [35, 36, 37, 38]. These conditions are useful for conceptual clarification because they may occur in relatively younger patients and can be considered with fewer confounding effects of aging, frailty, multimorbidity, and polypharmacy. By using these relatively distinct prototypes as conceptual anchors, clinicians can more clearly interpret the complex swallowing phenotypes observed in older adults, in whom neural and muscular contributors often overlap.

However, these prototypes are not representative of most dysphagia encountered in daily clinical practice. Severe bulbar dysphagia caused by focal brainstem lesions is clinically important but uncommon, and Duchenne muscular dystrophy is a rare disease. In contrast, dysphagia encountered across acute, post‐acute rehabilitation, geriatric, and long‐term care settings rarely presents as a purely neural control‐dominant or purely muscle function‐dominant disorder.

This mixed nature is often apparent on videofluoroscopy and endoscopic evaluation. Standardized videofluoroscopic frameworks assess swallowing across multiple physiological components, including swallow initiation, laryngeal vestibular closure, pharyngeal contraction, pharyngoesophageal segment opening, and pharyngeal residue [39]. These components are frequently abnormal in combination rather than in isolation. Delayed swallow initiation, premature bolus entry into the pharynx, or impaired airway protection may coexist with pharyngeal residue, reduced bolus propulsion, and inefficient clearance.

Importantly, pharyngeal residue itself is not a purely muscular sign. It may reflect reduced pharyngeal contraction, impaired tongue base retraction, inadequate laryngeal elevation, impaired UES opening, sensory impairment, bolus properties, or impaired coordination [39, 40]. Mixed instrumental findings should therefore not be reduced to a single dominant pathophysiological process. Delayed swallow triggering may coexist with weak pharyngeal clearance, and aspiration risk may be influenced by both impaired timing before or during the swallow and residual bolus after the swallow [39, 40, 41].

The value of the two prototypes lies not in classifying patients into exclusive categories, but in providing conceptual anchors for interpreting mixed instrumental findings. Neural control and muscle function represent important axes of dysphagia, but clinical swallowing disorders often arise from their interaction. This recognition provides a basis for understanding why dysphagia in clinical practice, particularly in older adults, commonly requires a broader multifactorial perspective that also considers activity level, systemic reserve, and external modifiers.

## 4 Dysphagia in Older Adults as a Geriatric Syndrome

Geriatric syndromes are generally understood as clinically important conditions in older adults that arise from the interaction of multiple risk factors, reduced physiological reserve, functional vulnerability, and environmental or care‐related factors [42]. They may coexist with major diseases such as stroke, cancer, heart failure, or infection, and they often modify clinical presentation, prognosis, and management needs without replacing the primary disease diagnosis.

The same structure can be applied to dysphagia in older adults. Disease‐based labels, such as post‐stroke dysphagia, Parkinson‐related dysphagia, or cancer‐related dysphagia, remain clinically meaningful because they identify important disease contexts. However, in older adults, these labels often do not fully explain why dysphagia emerges, persists, or worsens in a given patient. Swallowing dysfunction may be shaped by disease‐specific impairment together with age‐related vulnerability, reduced reserve, functional decline, malnutrition, reduced activity, medication effects, and care‐related factors [1, 2, 27, 29, 42].

Dementia‐associated dysphagia further illustrates this geriatric‐syndrome perspective. Swallowing difficulty in dementia may reflect not only impaired oropharyngeal physiology but also reduced attention, impaired recognition of food, feeding apraxia, dependence in feeding, behavioral symptoms, malnutrition, and care‐environment factors. These features make dementia‐associated dysphagia a clinically important example in which disease burden, cognitive and functional status, activity limitation, systemic vulnerability, and external modifiers interact [43, 44].

Accordingly, dysphagia in older adults can be understood as a geriatric syndrome when clinically apparent swallowing dysfunction emerges from the interaction of disease burden, physiological vulnerability, functional status, and contextual factors. This framing does not replace disease‐based diagnosis. Instead, it helps explain why patients with the same diagnostic label may follow different clinical courses and require different management strategies. It also provides a bridge from disease‐based diagnosis to mechanism‐based assessment by encouraging clinicians to identify interacting and potentially modifiable contributors that shape swallowing function.

## 5 From Disease‐Based Diagnosis to Mechanism‐Based Assessment

**FIGURE 1:**
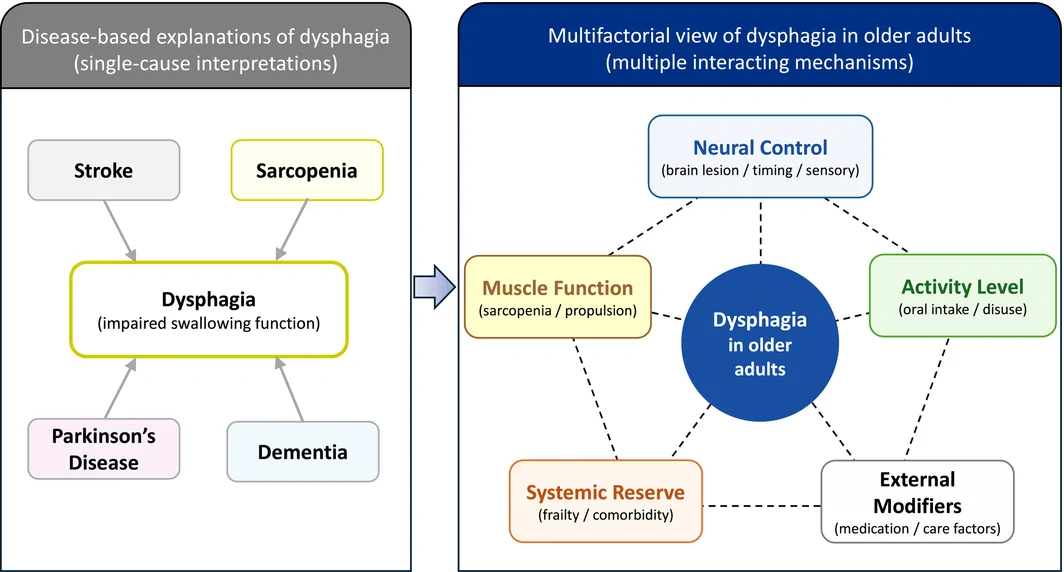
From disease‐based explanations to a multifactorial view of dysphagia in older adults. Contrasts disease‐based or single‐cause interpretations of dysphagia with a multifactorial view of dysphagia in older adults. In disease‐based explanations, dysphagia may be interpreted as a direct consequence of a single dominant factor or diagnostic label, such as stroke, sarcopenia, Parkinson's disease, or dementia. In older adults, swallowing dysfunction is often shaped by interacting domains, including neural control, muscle function, activity level, systemic reserve, and external modifiers. The figure illustrates the conceptual shift from a linear disease‐based explanation toward a multifactorial understanding of dysphagia in later life.

Disease‐based diagnosis remains essential in dysphagia management. Conditions such as stroke, neurodegenerative disease, neuromuscular disease, head and neck cancer, sarcopenia, and aspiration pneumonia provide important information about clinical context, prognosis, risk, and treatment priorities. However, as discussed above, disease labels alone may not fully explain the mechanisms that shape swallowing dysfunction in an individual patient. Disease‐based diagnosis should therefore be complemented by assessment of contributing mechanisms. This perspective integrates physiological mechanisms, functional activity, and contextual factors into a clinically oriented structure for geriatric dysphagia care (Figure 1).

A mechanism‐based approach first requires clarification of the level at which dysphagia is being assessed. From the perspective of the International Classification of Functioning, Disability and Health (ICF), swallowing can be evaluated at the level of body functions and structures as well as at the level of activities [45]. In a narrower physiological sense, oropharyngeal dysphagia refers to impairments in swallowing function that can be evaluated using instrumental assessments such as videofluoroscopic swallowing study (VFSS), FEES, manometry, surface electromyography, or tongue pressure measurement. These assessments can characterize physiological abnormalities, including delayed swallow initiation, impaired airway protection, pharyngeal residue, reduced bolus propulsion, impaired upper esophageal sphincter opening, and reduced tongue pressure.

In a broader clinical sense, dysphagia may also refer to difficulty maintaining safe, efficient, and adequate oral intake in daily life. This corresponds more closely to the ICF activity level. Measures such as FOIS and the Food Intake LEVEL Scale (FILS) provide activity‐level information on functional oral intake and feeding status [23, 46]. These measures are clinically important because they relate directly to nutrition, hydration, care burden, and patient outcomes. However, they do not necessarily identify the underlying physiological mechanism. Reduced oral intake may result from impaired swallowing physiology, but it may also reflect fatigue, cognition, poor appetite, oral health problems, inadequate feeding assistance, medical instability, or environmental factors. Thus, physiological impairment and activity limitation are related, but they are not interchangeable.

Within this framework, mechanism‐based assessment may be organized across five clinically relevant domains: neural control, muscle function, activity level, systemic reserve, and external modifiers. These domains are intended as a practical organizing structure rather than a fixed classification and should not be interpreted as a replacement for the ICF framework. Their number, boundaries, and relative weighting may be refined as further empirical evidence accumulates. They provide a practical way to organize mechanism‐based clinical reasoning within and across ICF‐related levels. Because some clinical observations, such as pharyngeal residue or reduced oral intake, may reflect more than one mechanism, Table 2 assigns each domain to its primary ICF‐related level and summarizes common findings, assessment examples, and potential clinical implications as an illustrative clinical mapping rather than a strict classification.

**TABLE 2:** Mechanism‐oriented clinical mapping of dysphagia contributors.

Domain	Primary ICF‐related level	Common findings or contributors	Assessment examples	Clinical implications
Neural control	Body functions and structures	Delayed swallow initiation; impaired coordination; reduced airway protection; sensory impairment	VFSS; FEES; neurological examination; consciousness and cognition assessment; sensory assessment	Sensory stimulation; texture modification for timing and bolus flow control; airway protection strategies; attention to alertness and cognition
Muscle function	Body functions and structures	Reduced bolus propulsion; reduced pharyngeal clearance; fatigue during swallowing; reduced tongue or pharyngeal force	VFSS; FEES; tongue pressure; sEMG; manometry where available	Nutrition support; resistance‐based rehabilitation; endurance training; texture modification for clearance
Activity level	Activities	Reduced functional oral intake; dietary modification; prolonged mealtime; feeding dependence; reduced swallowing use or meal participation	FOIS; FILS; dietary intake assessment; meal observation; assessment of rehabilitation participation	Graded oral intake; mealtime support; feeding assistance; promotion of swallowing use; rehabilitation planning
Systemic reserve	Health condition/personal vulnerability, with body function components	Frailty; malnutrition; comorbidities; respiratory dysfunction; inflammation; fatigue; reduced recovery capacity	Frailty assessment; nutritional assessment; respiratory evaluation; comorbidity review; physical function assessment	Nutrition support; medical optimization; respiratory management; physical rehabilitation
External modifiers	Environmental and care‐related factors	Medication effects; fasting policy; insufficient oral care; feeding environment; care practices	Medication review; care process review; oral health assessment; feeding environment assessment	Medication adjustment; oral management; care planning; interdisciplinary coordination

*Note:* Provides an illustrative mapping of five clinically relevant domains for mechanism‐based assessment: Neural control, muscle function, activity level, systemic reserve, and external modifiers. The t summarizes common findings, assessment examples, and potential clinical implications for each domain. It is intended to support clinical reasoning and interdisciplinary communication, not to serve as a validated assessment tool or fixed classification system.

Abbreviations: FEES, fiberoptic endoscopic evaluation of swallowing; FILS, Food Intake Level Scale; FOIS, Functional Oral Intake Scale; sEMG, surface electromyography; VFSS, videofluoroscopic swallowing study.

Neural control and muscle function mainly correspond to body functions and structures. Neural control includes brain lesions, consciousness level, sensory input, swallow triggering, coordination, and airway protection. Muscle function includes tongue and pharyngeal force, bolus propulsion, clearance, and fatigability during swallowing. Importantly, a disease label does not necessarily specify which of these mechanisms predominates. For example, dysphagia labeled as sarcopenic dysphagia may involve muscle dysfunction as an important contributor, but reduced bolus propulsion alone may not fully explain the clinical presentation. Neural or sensorimotor features, such as delayed swallow initiation, sensory impairment, or impaired airway protection, may also be present together with reduced activity or systemic vulnerability.

The remaining domains extend assessment beyond swallowing physiology. Activity level corresponds primarily to the ICF activity domain and includes oral intake pattern, swallowing use, mealtime performance, dietary restriction, and participation in rehabilitation. Systemic reserve does not map to a single ICF component. It reflects the individual's health condition and personal vulnerability, including frailty, malnutrition, comorbidities, respiratory status, inflammation, fatigue, and recovery capacity. External modifiers correspond mainly to environmental and care‐related factors, including medications, treatment decisions, fasting policies, feeding assistance, oral care, care environment, and other iatrogenic or contextual influences.

This five‐domain perspective helps clinicians identify dominant and modifiable contributors to dysphagia. A patient with delayed swallow initiation may require attention to neural control and sensory input, whereas a patient with marked residue and fatigue may require assessment of muscle function, nutrition, and systemic reserve. Reduced oral intake may reflect activity limitation, but the relevant mechanism may lie in any combination of neural, muscular, systemic, activity‐related, or environmental factors. The goal is not to assign a patient to a single domain, but to clarify which contributors are most relevant and which can be modified.

This approach naturally supports comprehensive and multidisciplinary management. Dysphagia in older adults often involves swallowing physiology, nutrition, physical function, cognition, medication, oral health, respiratory status, and care environment. Physicians, rehabilitation professionals, nurses, dietitians, pharmacists, dentists, or oral health professionals, and care staff may each contribute to identifying and modifying relevant factors. By complementing disease‐based diagnosis with mechanism‐based assessment, clinicians may better understand the clinical reality of dysphagia in older adults and select interventions that are more individualized, comprehensive, and clinically meaningful.

**FIGURE 2:**
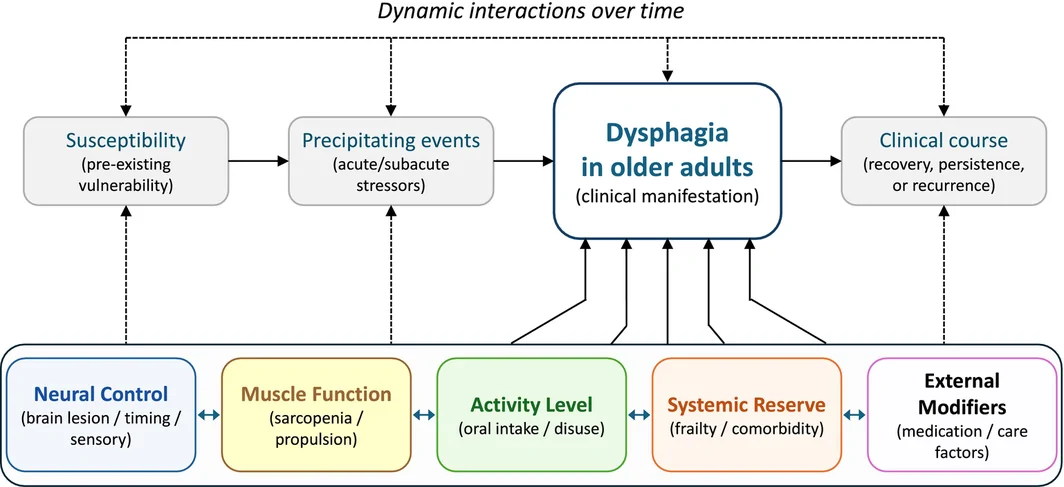
Dysphagia in older adults as a dynamic, multifactorial, and interaction‐driven condition. Illustrates dysphagia in older adults as a dynamic condition influenced by interactions among susceptibility, precipitating events, dysphagia emergence, and clinical course. Across these stages, neural control, muscle function, activity level, systemic reserve, and external modifiers may interact over time and influence the onset, persistence, recovery, or recurrence of dysphagia. The figure emphasizes that dysphagia in older adults is a dynamic multifactorial process shaped by changing physiological, functional, and care‐related factors. The framework is intended to support clinical reasoning by highlighting interacting and potentially modifiable contributors rather than defining fixed categories.

In daily geriatric practice, this five‐domain perspective can be operationalized as a simple clinical reasoning pathway: first, identify the primary disease‐based diagnosis; second, screen for dominant and modifiable contributors across the five domains, such as neural timing delay, reduced muscle output, activity limitation, reduced systemic reserve, or external modifiers; and third, prioritize targeted multidisciplinary interventions according to the resulting mechanism profile. This pathway is not intended as a scoring system or validated tool, but as a practical guide for structuring assessment and team communication (Figure 2).

## 6 Future Perspective: Toward Mechanism‐Based Dysphagia Profiling

The five‐domain structure described above is intended as a practical way to organize clinical reasoning, not as a fixed or validated classification system. Future studies are needed to determine whether these domains are sufficient, whether some domains should be combined or subdivided, and how their relative contributions should be assessed in different clinical populations. The structure may ultimately require refinement as empirical evidence accumulates.

A future direction is the development of structured approaches to describe the mechanism profile of dysphagia in older adults. Such profiling could help clinicians summarize the dominant and modifiable contributors to swallowing dysfunction while preserving disease‐based diagnoses, instrumental findings, and activity‐level measures. Patients with similar oral intake levels may have different underlying profiles of neural control, muscle function, systemic reserve, activity limitation, or external modifiers. Conversely, patients with the same disease label may require different management strategies depending on their mechanism profile.

Mechanism‐based profiling may also support interdisciplinary communication and research classification. Dysphagia management in older adults often involves multiple professionals, and a shared description of relevant mechanisms may help clarify why particular interventions are selected and which domains require monitoring. In research, profiling could help characterize heterogeneous populations more precisely and may facilitate comparison across studies.

At present, this remains a conceptual direction. A framework for dysphagia mechanism assessment and profiling may be useful in the future, but it should be regarded as a possible approach requiring further development, consensus‐building, and validation, not as an established clinical tool.

## 7 Conclusion

Dysphagia in older adults is best understood as a multifactorial clinical condition that shares key characteristics of geriatric syndromes. Disease‐based diagnosis remains an important starting point, but it may not fully explain the clinical presentation, course, or management needs of older patients with dysphagia. In daily practice, clinicians often recognize this complexity: even when findings overlap, two patients with the same diagnostic label may require different approaches when one presents mainly with delayed swallow initiation and the other with marked pharyngeal residue and fatigue.

This perspective does not necessarily introduce a completely new way of thinking. Many clinicians may sense, through repeated assessment and management, that dysphagia in older adults is rarely explained by a single diagnosis or mechanism. The value of the present framework lies in making this implicit clinical reasoning more explicit, structured, and shareable across disciplines.

By organizing dysphagia in older adults as a multifactorial geriatric syndrome, clinicians may better identify dominant and modifiable contributors, integrate physiological and activity‐level assessments, and support more individualized, comprehensive, and multidisciplinary management in geriatric and rehabilitation practice.

## Author Contributions

Hideaki Takahata conceived the review, drafted the manuscript, prepared the tables and figures, critically revised the manuscript, and approved the final version.

## Funding

The author has nothing to report.

## Ethics Statement

The author has nothing to report.

## Consent

The author has nothing to report.

## Conflicts of Interest

The author declares no conflicts of interest.

## Data Availability

Data sharing not applicable to this article as no datasets were generated or analysed during the current study.
